# Registration of Brain MRI/PET Images Based on Adaptive Combination of Intensity and Gradient Field Mutual Information

**DOI:** 10.1155/2007/93479

**Published:** 2007-03-20

**Authors:** Jiangang Liu, Jie Tian

**Affiliations:** ^1^Medical Image Processing Group, Key Laboratory of Complex Systems and Intelligence Science, Institute of Automation, Chinese Academy of Science, P.O. Box 2728, Beijing 100080, China; ^2^Life Science Center, Xidian University, Xi'an, Shaanxi 710071, China

## Abstract

Traditional mutual information (MI) function aligns two multimodality images with intensity information, lacking spatial information, so that it usually presents many local maxima that can lead to inaccurate registration. Our paper proposes an algorithm of adaptive combination of intensity and gradient field mutual information (ACMI). Gradient code maps (GCM) are constructed by coding gradient field information of corresponding original images. The gradient field MI, calculated from GCMs, can provide complementary properties to intensity MI. ACMI combines intensity MI and gradient field MI with a nonlinear weight function, which can automatically adjust the proportion between two types MI in combination to improve registration. Experimental results demonstrate that ACMI outperforms the traditional MI and it is much less sensitive to reduced resolution or overlap of images.

## 1. INTRODUCTION

Multimodal image registration plays a significant role in medical image processing such as medical diagnosis, therapeutic planning and assessment [1]. MI has proved to be an effective criterion for the multimodal medical image registration [1–3]. However, even with this method, the correct alignment cannot be guaranteed, especially when it is applied to images with low resolution or small overlapped area. MI function traditionally relies on only intensity information of images, lacking sufficient spacial information, so it has difficulty in accurately measuring the degree of alignment of two images. It is also apt to be influenced by intensity interpolation, therefore presents many local maxima which frequently lead to misregistration [4, 5].

Different tissues in human brain usually present different gray intensity no matter which imaging modality is applied to them. The intensity gradient at the transition of two tissues is steeper than the interior, where the gradient magnitude and phase lie on the imaging modality, and the spatial relative position is invariable. Therefore the gradient fields of two images can provide effective spatial information for their similarity measurement.

Some research introduced gradient or feature information into multimodal image registration to improve the performance of registration function. Butz and Thiran [6] performed the registration with MI based on feature space; Pluim et al. [7] integrated gradient information into mutual information to get a relatively smooth registration function; Haber and Modersitzki [8] presented an alternative similarity measurement based on normalized gradient field for multimodal image registration; Maintz et al. [9] showed that image intensity gradient was an effective multimodal image registration criteria. These methods were effective for the improvement of registration quality. Our work took full advantage of the gradient phase information and the relationship between intensity images and their gradient fields to further improve the performance of MI function.

Our current study proposes a technique for Multimodal image registration, namely adaptive combination of intensity and gradient field mutual information (ACMI). Unlike the intensity MI computed with original images, the gradient field MI is calculated with gradient code maps (GCM) which were obtained from corresponding original images by a spherical gradient coder. The intensity of each point in GCM is completely determined by the gradient vector of corresponding point in original intensity image, so that the magnitude and phase information of spatial gradient field of original image is converted into intensity information of GCM. The properties of these two MI functions are complementary for each other and the ACMI is defined as the sum of products of each MI function and corresponding weighting function. The weighting function can be adaptively regulated to highlight the contribution of MI function with better performance to ACMI.

The simulated data experiment and the actual registration experiment were conducted to compare the performance of ACMI and traditional MI. The results of simulated data experiment showed that ACMI function was much smoother and more reliable than traditional MI. The statistical test for the results of actual registrations demonstrated that the registration quality with ACMI was significantly higher than that with traditional MI and it was much less sensitive to the reduction of resolution or overlapped region of images.

## 2. METHODS

### 2.1. Mutual information

Given reference image *R* and floating image *F* with their respective marginal intensity distributions *p_R_*, *p_F_* and joint intensity distribution *p_RF_*, their MI is defined by means of Kullback-Leibler measure [3]:

(1) $$I\left(R,F\right)=\sum_{i,j}p_{RF}\left(i,j\right)\log\frac{p_{RF}\left(i,j\right)}{p_{R}\left(i\right)p_{F}\left(j\right)}$$.

The MI criterion postulates that the images are geometrically aligned when *I*(*A, B*) is maximal. However, this is not always true, because many local maxima are frequently announced and sometimes even worse, the global maximum does not correspond to the correct alignment [5].

MI is also defined by means of information theoretic notion of entropy. Given images *R* and *F* with their respective entropies *H*(*R*) and *H*(*F*) and their joint entropy *H*(*R, F*), their MI *I*(*R, F*) is defined as

(2) I(R,F)=H(R)+H(F)−H(R,F).

In fact, MI is a measure method based on statistical notion, and its reliability depends on the number of voxels included in its computation. It is sensitive to image resolution or the overlapped area of two images. To solve this problem, some MI-related measures have been proposed such as entropy correlation coefficient (ECC) *E*(*R, F*) [10]

(3) $$E\left(R,F\right)=\frac{2I\left(R,F\right)}{H\left(R\right)+H\left(F\right)}$$

and normalized mutual information NMI *N*(*R, F*) [11]

(4) $$N\left(R,F\right)=\frac{H\left(R\right)+H\left(F\right)}{H\left(R,F\right)}$$.

The same artifact patterns as MI function are found in both ECC and NMI [4]. It does not guarantee an accurate and reliable alignment. Comparing (2) and (3), for fixed images *R* and *F*, their respective entropies *H*(*R*) and *H*(*F*) are approximately constant, so the EEC is in fact the product of *I*(*R, F*) and a constant. As a result, ECC has the similar performance to MI except for its normalized value range [0, 1] [1]. Therefore in the following analysis, ECC is used in place of corresponding MI.

### 2.2. Spatial gradient field code

Given a 2D image *F* with intensity *f* (*x, y*), its spatial gradient field *G_F_* (*x, y*) can be computed by

(5) $$G_{F}\left(x,y\right)=\frac{\partial f\left(x,y\right)}{\partial x}\vec{i}+\frac{\partial f\left(x,y\right)}{\partial y}\vec{j}$$,

where the $\vec{i}$ and $\vec{j}$ are the unit vectors along *x* and *y* axes, respectively. If the horizontal and vertical derivatives, namely *∂f* (*x*, *y*)/*∂x* and *∂f* (*x*, *y*)/*∂ y*, are denoted by *f_x_* and *f_y_*, respectively, then the magnitude *ρ_i, j_* and phase *θ_i, j_* ([0, 2*π*)) of gradient of voxel *f_i, j_* are calculated by

(6) $$\begin{array}{c} \gamma_{i,j}=\sqrt{f_{x}^{2}+f_{y}^{2},}\\ \gamma_{\max}=\max\left(\gamma_{i,j}\right),\\ \rho_{i,j}=\{\begin{array}{ll} \frac{\gamma_{i,j}}{\gamma_{\max}} & \gamma_{i,j}<\gamma_{\max},\\ 0.999 & \gamma_{i,j}=\gamma_{\max}, \end{array}\\ \theta_{i,j}=\{\begin{array}{ll} \tan^{-1}\frac{f_{y}}{f_{x}} & f_{x}>0,f_{y}>0,\\ \tan^{-1}\frac{f_{y}}{f_{x}}+\pi & f_{x}<0,\\ \tan^{-1}\frac{f_{y}}{f_{x}}+2\pi & f_{x}>0,f_{y}<0,\\ \frac{\pi }{2} & f_{x}=0,f_{y}>0,\\ \frac{3\pi }{2} & f_{x}=0,f_{y}<0. \end{array} \end{array}$$

**Figure 1:**
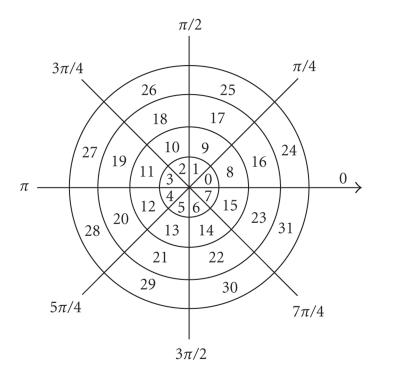
Illustration of a 2D gradient coder (Δ_*ρ*_ = 1/4, Δ_*θ*_ = *π*/4).

The ranges of *ρ_i, j_* and *θ_i, j_* are [0, 1) and [0, 2*π*), respectively. The point *c_i, j_* in GCM *C* is obtained by coding the gradient vector (*ρ_i, j_*, *θ_i, j_*) of corresponding point *f_i, j_* in *F* with gradient coder [12],

(7) $$c_{i,j}=\{\begin{array}{ll} \left\lfloor \frac{\rho_{i,j}}{\Delta_{p}}\right\rfloor N+\left\lfloor \frac{\theta_{i,j}}{\Delta_{\theta }}\right\rfloor & \rho_{i,j}\geq \text{Th,}\\ \text{0} & \rho_{i,j}<\text{Th}, \end{array}$$

where ⌊*ρ_i, j_*/Δ*_p_*⌋ is the integer portion of *ρ_i, j_*/Δ*_p_* and Th is a prespecified threshold to ignore the point with low gradient magnitude. Δ*_ρ_* and Δ*_θ_* are, respectively, the magnitude and phase bin intervals of gradient coder, and *N* equals to 2*π*/Δ_*θ*_. Figure 1 illustrates a 2D gradient coder. It converts gradient difference of points in gradient field into intensity difference of points in GCM. Given two gradient vectors with equal magnitude, the one with more phase (*θ_i, j_*) will produce stronger intensity in GCM. Table 1 shows an example of mapping from gradient field of intensity image to GCM. The most left column and the top row of Table 1 correspond to phase angle and magnitude of gradient vector, respectively. For example, two pixels with gradient vectors (0.3, *π*/8) and (0.3, 5*π*/8) will produce intensity values 8 and 10 in GCM, respectively. The gradient field map includes both magnitude and phase information, so it can provide more spatial information for the similarity measurement of two images.

**Figure 2:**
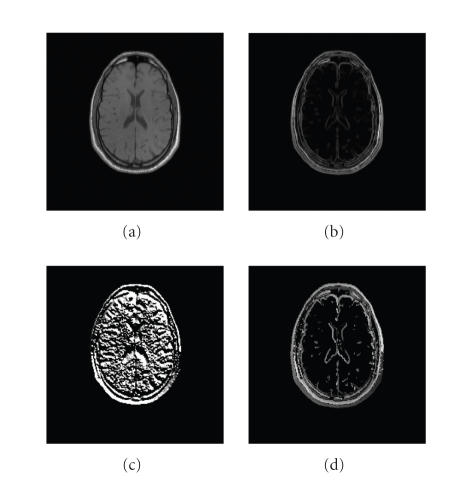
A slice of 3D GCM of MRI T1. (a) Original image, (b) gradient magnitude map, (c) gradient phase map, (d) GCM (Δ_*ρ*_ = 1/16, Δ_*θ*_ = *π*/8, Δ_*φ*_ = *π*/8, and Th = 0.10).

**Figure 3:**
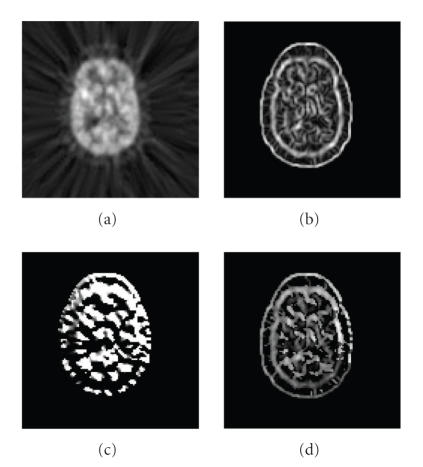
A slice of 3D GCM of PET. (a) Original image, (b) gradient magnitude map, (c) gradient phase map, (d) GCM (Δ_*ρ*_ = 1/16, Δ_*φ*_ = *π*/8, Δ_*θ*_ = *π*/8, and Th = 0.16).

This gradient coder can be easily extended to 3D images. Given a voxel *f_i, j, k_* of 3D image *F* with gradient vectors (*f_x_*, *f_y_*, *f_z_*), the 3D gradient coder is defined as

(8) $$c_{i,j,k}=\{\begin{array}{ll} \left\lfloor \frac{\rho_{i,j,k}}{\Delta_{p}}\right\rfloor NK+\left\lfloor \frac{\varphi_{i,j,k}}{\Delta_{\varphi }}\right\rfloor K+\left\lfloor \frac{\theta_{i,j,k}}{\Delta_{\theta }}\right\rfloor & \rho_{i,j,k}\geq \text{Th},\\ \text{0} & \rho_{i,j,k}<\text{Th}, \end{array}$$

with

(9) $$\begin{array}{c} \gamma_{i,j,k}=\sqrt{f_{x}^{2}+f_{y}^{2}+f_{z}^{2},}\\ \gamma_{\max}=\max\left(\gamma_{i,j,k}\right),\\ \rho_{i,j,k}=\{\begin{array}{ll} \frac{\gamma_{i,j,k}}{\gamma_{\max}} & \gamma_{i,j,k}<\gamma_{\max},\\ 0.999 & \gamma_{i,j,k}=\gamma_{\max}, \end{array}\\ \varphi_{i,j,k}=\cos^{-1}\frac{f_{z}}{\gamma_{i,j,k}},\\ \theta_{i,j,k}=\{\begin{array}{ll} \tan^{-1}\frac{f_{y}}{f_{x}} & f_{x}>0,f_{y}>0,\\ \tan^{-1}\frac{f_{y}}{f_{x}}+\pi & f_{x}<0,\\ \tan^{-1}\frac{f_{y}}{f_{x}}+2\pi & f_{x}>0,f_{y}<0,\\ \frac{\pi }{2} & f_{x}=0,f_{y}>0,\\ \frac{3\pi }{2} & f_{x}=0,f_{y}<0, \end{array} \end{array}$$

where *φ_i, j, k_*([0, *π*]) and *θ_i, j, k_*([0, 2*π*)) are the polar angle and azimuthal angle, respectively. *N* and *K* equal to *π*/Δ_*φ*_ and 2*π*/Δ_*θ*_, respectively, where Δ_*φ*_ and Δ_*θ*_ are the polar angle and azimuthal angle bin intervals of 3D gradient coder, respectively. The other notations are defined similarly as in (6). With (8), spatial gradient field (magnitude and phase) information of original images is converted into intensity information of GCMs. Figures 2 and 3 show a slice of GCMs of 3D MRI T1 and PET images, respectively.

### 2.3. Adaptive combination of intensity and gradient field mutual information (ACMI)

With (3), the intensity ECC *E_i_* is obtained from two original images and the gradient ECC * E_g_* is computed from their GCMs. The ACMI *E_a_* is defined as

(10) $$E_{a}=f\left(v\left(E_{i},E_{g}\right)\right)E_{i}+\left(1-f\left(v\left(E_{i},E_{g}\right)\right)\right)E_{g}$$,

with

(11) $$\begin{array}{c} f\left(v\left(E_{i},E_{g}\right)\right)=\frac{1}{1+\exp\left(-\left(v\left(E_{i},E_{g}\right)-0.5\right)/T\right)},\\ v\left(E_{i},E_{g}\right)=\frac{E_{i}+E_{g}}{2}\;\;\;\;0\leq E_{i},\;\;\;E_{g}\leq 1. \end{array}$$

**Figure 4:**
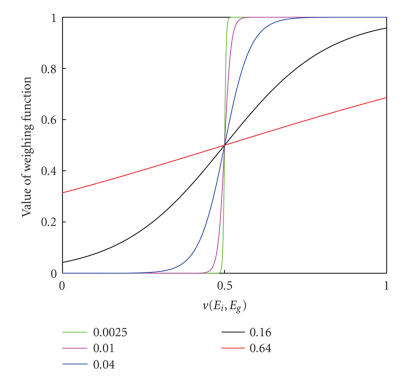
Weighing functions *f* (*v*(*E_i_*, *E_g_*)) with *T* = 0.0025 (green), *T* = 0.01 (magenta), *T* = 0.04 (blue), *T* = 0.16 (black) and *T* = 0.64 (red), respectively.

As shown in Figure 4, the weighting function *f* (*v*(*E*_i_ **, *E_g_*)) actually is a logistic function with rightward half unit shift. This function has some expected properties [13]. The first is the saturation with the maximum of one and the minimum of zero. This property is very important for the weighting function because, as described by (10), the output of *f* (*v*(*E*_i_ **, *E_g_*)) presents a fraction whose value extends from zero to one. The second is differentiability which not only prevents introducing additional local maxima during combination of registration functions but also facilitates the optimization of ACMI with some derivative-needed techniques such as Gauss-Newton optimization method. The third, the most important one, is the nonlinearity. As indicated in Figure 4, the weighting function presents nonlinear characteristic in two terminal saturating parts but approximate linear characteristic in the middle nonsaturating part. Thus, according to (10), the ACMI is mostly determined by one of two ECC functions at each nonlinear terminal (gradient ECC for the left terminal and intensity ECC for the right), but equals to the sum of two ECC functions with similar weights in the middle linear part. The nonlinear degree of weighting function totally depends on the time constant *T*. If it is too large, for example 0.64 (Figure 4, the red line), the weighting function will present more linear characteristic. As a result, the unexpected middle linear part is lengthened and the weight-similar sum of registration functions can lead to severe roughness of ACMI. Decrease of time constant can shorten this unexpected middle linear part and lengthen the terminal saturating parts. On the other hand, the extremely small time constant, for example 0.0025 (Figure 4, green line), can impair the differentiability of weighting function and introduce additional local maxima when combining two registration functions. The optimal choice for *T* was obtained by experimental method (described in Section 3). In terms of these three properties, *f* (*v*(*E*_i_ **, *E_g_*)) is a desirable weighting function for combination of registration functions.

As shown in Figures 2(a) and 3(a), the original images contain abundant information. It has two effects on their ECC function. On the one hand, the similarity measurement of two images is more reliable because of abundant information and the ECC function presents a tendency of convergence to the global maximum which corresponds to the correct alignment. On the other hand, abundant information means strong nonuniformity of intensity across voxels, then the ECC function is easily influenced by intensity interpolation, and thereby presents many local maxima which can lead to inaccurate registration (see Figures 6(a), 6(d), and 6(g)).

Compared to the original images, as shown in Figures 2(d) and 3(d), GCMs contain less information (most voxels have zero intensity value except those at edges of some tissues). This relatively higher-intensity uniformity reduces the effect of intensity interpolation on ECC function of two maps and therefore makes it smoother [4]. Additionally the edge information in GCMs can provide reliable and accurate spatial information for the similarity measurement of images. However, in the neighborhood of global maximum, the ECC function often presents plateaus or valleys, preventing convergence to global maximum (see Figures 6(b), 6(e), and 6(h)).

According to their complementary properties, these two ECC functions are combined by ACMI, using a nonlinear weighting function (10). In our study, the downhill simplex optimization [14] was used for the search of maximum in six-dimensional space (translations along *x*, *y*, and *z* axes, and rotations around *x*, *y*, and *z* axes). This algorithm is an efficient method for *N*-dimensional unconstrained minimization [15, 16]. It begins with *N* + 1 vertices which define a simplex in *N*-dimensional space and attempts to move them into the minimum. Given reference image *R*, floating image *F*, and transforming parameter vector (vertex) *x_j_* ^(*k*)^ (*j* = 1, 2,…, 7) in *k*th iteration, *TF*(*x_j_* ^(*k*)^) denotes the transformed *F* with *x_j_* ^(*k*)^, and *Ei_j_* ^(*k*)^, *Eg_j_* ^(*k*)^ and *Ea_j_* ^(*k*)^ denote intensity ECC, gradient ECC and ACMI of *R*, and *TF*(*x_j_* ^(*k*)^), respectively. The iterative procedure is the following [16].

*Step 1.* Initialize *x_j_* ^(*k*)^ and calculate *Ea_j_* ^(*k*)^.

*Step 2.* Order *x_j_* ^(*k*)^ to satisfy *Ea_1_* ^(*k*)^ ≤ *Ea_2_* ^(*k*)^ ≤ ⋯ ≤ *Ea_7_* ^(*k*)^, and calculate the centroid of the six best ACMI values, ${\bar{x}}^{\left(k\right)}=\sum_{j=1}^{6}x_{j}^{\left(k\right)}/6$ and ${\bar{Ea}}^{k}$.

*Step 3.* $x_{r}^{\left(k\right)}={\bar{x}}^{\left(k\right)}+\left({\bar{x}}^{\left(k\right)}-x_{7}^{\left(k\right)}\right)$ and calculate *Ea_r_* ^(*k*)^.

*Step 4.* If *Ea_1_* ^(*k*)^ ≤ *Ea_r_* ^(*k*)^ < *Ea_6_* ^(*k*)^, then *x_7_* ^(*k*)^ = *x_r_* ^(*k*)^, *Ea_7_* ^(*k*)^ = *Ea_r_* ^(*k*)^ and go to Step 9.

*Step 5.* If *Ea_r_* ^(*k*)^ < *Ea_1_* ^(*k*)^, then $x_{e}^{\left(k\right)}={\bar{x}}^{\left(k\right)}+2\left(x_{r}^{\left(k\right)}-{\bar{x}}^{\left(k\right)}\right)$, and calculate *Ea_e_* ^(*k*)^. If *Ea_e_* ^(*k*)^ < *Ea_r_* ^(*k*)^, then *x_7_* ^(*k*)^ = *x_e_* ^(*k*)^, *Ea_7_* ^(*k*)^ = *Ea_e_* ^(*k*)^, and go to Step 9; otherwise *x_7_* ^(*k*)^ = *x_r_* ^(*k*)^, *Ea_7_* ^(*k*)^ = *Ea_r_* ^(*k*)^, and go to Step 9.

*Step 6.* If *Ea_6_* ^(*k*)^ ≤ *Ea_r_* ^(*k*)^ < *Ea_7_* ^(*k*)^, then $x_{c}^{\left(k\right)}={\bar{x}}^{\left(k\right)}+0.5\left(x_{r}^{\left(k\right)}-{\bar{x}}^{\left(k\right)}\right)$, and calculate *Ea_c_* ^(*k*)^. If *Ea_c_* ^(*k*)^ ≤ *Ea_r_* ^(*k*)^, then *x_7_* ^(*k*)^ = *x_c_* ^(*k*)^, *Ea_7_* ^(*k*)^ = *Ea_c_* ^(*k*)^, and go to Step 9; otherwise go to Step 8.

*Step 7.* If *Ea_r_* ^(*k*)^ ≥ *Ea_7_* ^(*k*)^, then $x_{cc}^{\left(k\right)}={\bar{x}}^{\left(k\right)}+0.5\left({\bar{x}}^{\left(k\right)}-x_{7}^{\left(k\right)}\right)$ and calculate *Ea_cc_* ^(*k*)^. If *Ea_cc_* ^(*k*)^ < *Ea_7_* ^(*k*)^, then *x_7_* ^(*k*)^ = *x_cc_* ^(*k*)^, *Ea_7_* ^(*k*)^ = *Ea_cc_* ^(*k*)^, and go to Step 9; otherwise, go to Step 8.

*Step 8.* *x_j_* ^(*k*)^ ← *x_j_* ^(*k*)^ + 0.5(*x_1_^k^* − *x_j_* ^(*k*)^).

*Step 9.* $c={\left\{\left(1/7\right)\sum_{j=1}^{7}{\left[Ea_{j}^{\left(k\right)}-{\bar{Ea}}^{\left(k\right)}\right]}^{2}\right\}}^{1/2}$. If *c* < 10^−4^, then the iteration exists; otherwise *k* ← k + 1, and go to Step 2.

In each iteration, the ACMI *Ea_j_* ^(*k*)^ for each transforming parameter vector *x_j_* ^(*k*)^ is calculated as follows.

*Step 1.* Transform *F* into *TF*(*x_j_* ^(*k*)^) with transforming parameters vector *x_j_* ^(*k*)^.

*Step 2.* Calculate intensity ECC *Ei_j_* ^(*k*)^ and gradient ECC *Eg_j_* ^(*k*)^ of *R* and *TF*(*x_j_* ^(*k*)^).

*Step 3.* Adjust weighting function according to *Ei_j_* ^(*k*)^ and *Eg_j_* ^(*k*)^.

*Step 4.* Calculate ACMI *Ea_j_* ^(*k*)^.

When alignment improves, ACMI uses an iterative algorithm to automatically adjust the proportion between intensity ECC and gradient ECC by changing the weighting function. Thus at the coarse registration stage, the ACMI depends mostly on gradient ECC due to the low sum of ECC values and presents a smooth property facilitating the convergence to the basin of global maximum. With the two images increasingly aligned, the *v*(*E_i_*, *E_g_*) becomes larger due to the increase of the values of intensity ECC *E_i_* and gradient ECC *E_g_* (10). Accordingly, as indicated in Figure 4 (blue line), the weighting function shifts from the left saturating terminal to the right. At the fine stage where the gradient ECC varies slightly, the ACMI is determined mostly by intensity ECC for which the gradient ECC is a supplement. The higher the sum of ECCs is, the more reliable the intensity ECC is, therefore the more the ACMI depends on it than on gradient ECC. This coarse-to-fine and gradient-to-intensity strategy facilitates the convergence to global maximum which corresponds to correct alignment.

## 3. RESULTS

The brain image set used in the current study includes 35 brain MRI/PET image pairs (MRI T1, PD, T2, and their respective rectified versions versus PET). The brain image set and the standard transformations of each image pair were provided as the part of the project, “*Retrospective Image Registration Evaluation,*” Vanderbilt University, Nashville, TN [17]. The accuracy of each registration was evaluated by bone-marker-based gold standard, and the registration error was defined as the error distances between the gold standard in the reference image and the centroid of volume of interest (VOI) in the floating image after alignment (see [17] for more details). To compare performance of traditional ECC and ACMI for image pairs with low resolution or small overlapped area, two types of image pairs were generated from each original image pairs, namely subsampled version (subsampled by a factor of two in three axes, resp.) and small-overlapped version (50% overlapped region of original pairs).

In our study, the threshold Th for each type image is 0.10 for MRI T1, 0.08 for MRI T2, 0.12 for MRI PD, and 0.16 for PET. The thresholds were obtained by the basic global thresholding mehod [18].

As indicated by (8), the smaller the bin intervals of magnitude and phase of gradient coder are, the more gray levels the GCM has, and accordingly the more accurate registration result will be obtained. However, the increase of gray levels can decrease the statistic power of joint histogram from which the mutual information of two GCMs is calculated [4, 5]. Usually the overmuch gray levels of GCM are subsampled with a suitable bin interval width when calculating the joint histogram. So the extremely small bin intervals of magnitude and phase of gradient coder cannot improve the quality of registration. In our study, the magnitude, polar, and azimuthal angle bin intervals (Δ_*ρ*_, Δ_*φ*_, and Δ_*θ*_) are 1/16, *π*/8, and *π*/8, respectively. Thus the gray level of GCM is 2048 (16 × 8 × 16) which is enough to identify the changes of magnitude and phase of intensity images, and it is subsampled to 128 gray levels in the calculation of joint histogram.

**Figure 5:**
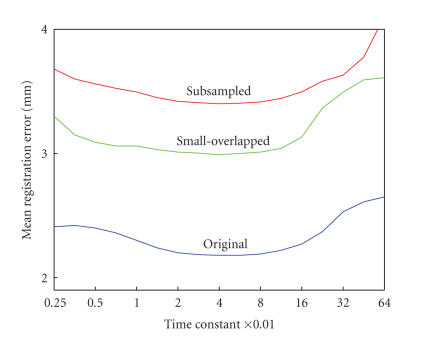
The mean registration error (RE) versus time constant for MRI/PET image pairs (blue), subsampled version (red) and small-overlapped version (green). The registration error is defined as the error distances between the gold standard in the reference image and the centroid of volume of interest (VOI) in the floating image after alignment. The horizontal axis is labeled with intervals of log_2_. For each image version, the registration error reaches the minimum near the time constant 0.04 where each is relatively insensitive to the changes of time constant.

The optimal choice of time constant was obtained by “bootstrap” method. Fifty “bootstrap” data sets were created by randomly selecting 20 MRI/PET image pairs 50 times with replacement from the brain image set. For a given “bootstrap” data set, registration was applied to each image pair using ACMI with each of 17 different time constants (from 0.0025 to 0.64). For a given time constant *T_j_*, the mean registration error across all “bootstrap” data sets was obtained by $\theta_{j}=\left(1/50\right)\sum_{i=1}^{50}m_{ji}$, where *m_ji_* denoted the median registration error across image pairs in *D_i_* for *T_j_*. Figure 5 shows mean registration error across all “bootstrap” data sets versus time constant for MRI/PET image pairs (blue), and their subsampled version (red) and small-overlapped version (green). As presented by Figure 5, mean registration error for each image version reaches the minimum near the time constant 0.04 where it is relatively insensitive to changes in the time constant.

**Figure 6:**
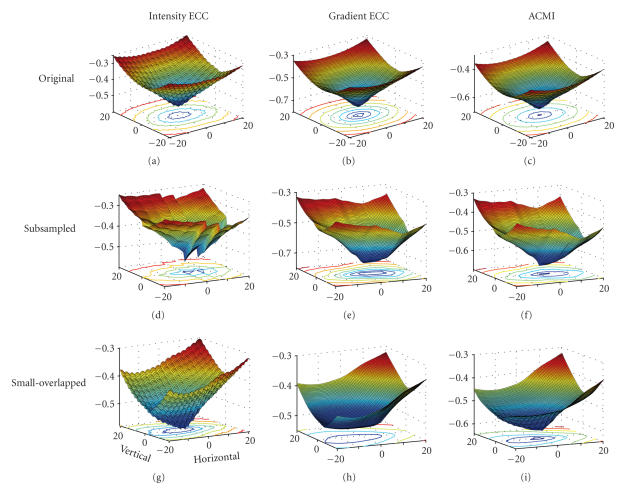
Registration function (PET/MRI T1) versus relative displacement between reference and floating images in horizontal and vertical orientations. For the original images, the ACMI performs slightly better than intensity and gradient ECC (top row). For the subsampled version, the intensity ECC presents a global maximum corresponding to the correct alignment, but also presents many local maxima, especially a secondary maximum near the global maximum (d). The gradient ECC presents less local maxima, but a valley at the bottom (e). For small-overlapped version, the intensity ECC is strongly rough, though it presents only a global maximum corresponding to the correct alignment (g). The gradient ECC is smoother, but presents a plateau at bottom not including the correct registration point (h). ACMI of each image version provides sufficient smoothness in the coarse registration stage and presents only one global maximum at coordinate (0, 0) of the graph (corresponding to the correct alignment) in the fine registration stage ((c), (f), and (i)).

Figure 6 describes three types of registration functions of a PET/MRI T1 pair, namely intensity ECC (traditional ECC), gradient ECC, and their adaptive combination ACMI versus relative displacements between reference and floating images in horizontal and vertical orientations. The negative registration functions are displayed for visual convenience. For the original images, the ACMI performs slightly better than intensity and gradient ECC (top row of Figure 6). For the subsampled version, the intensity ECC presents a global maximum corresponding to the correct alignment, but it still has many local maxima, especially a secondary maximum near the global maximum (Figure 6(d)). The gradient ECC presents less local maxima, but a valley at the bottom (Figure 6(e)). For small-overlapped version, the intensity ECC is strongly rough, though it presents only a global maximum corresponding to the correct alignment (Figure 6(g)). The gradient ECC is smoother, but presents a plateau at bottom not including the correct registration point (Figure 6(h)). Even the global optimization method such as simulated annealing or genetic algorithm is applied to these versions, the correct alignment is not guaranteed. ACMI overcomes these problems (Figures 6(f) and 6(i)). It provides sufficient smoothness in the coarse registration stage due to the dominance of gradient ECC. On the other hand, it presents only one global maximum at coordinate (0, 0) of the graph (corresponding to the correct alignment) in the fine registration stage because of the combination of larger part of intensity information (intensity ECC).

For each image pair, the registrations were applied to its three types of versions with traditional ECC, gradient ECC, and ACMI, respectively. Table 2 summarizes the results of registration. The values in the left three columns labeled with “Accuracy” are the median/maximal registration error for intensity ECC, gradient ECC, and ACMI, respectively, and the values in the right columns labeled with “Number of Iterations” are the mean and standard deviation of number of iterations (Mean ± SD) for intensity ECC, gradient ECC, and ACMI, respectively.

Relative to intensity ECC and gradient ECC, ACMI provides 20.7% and 11.3% mean reduction in error for original version, 19.6% and 8.1% for subsampled version, and 22.5% and 10.8% for small overlapped version, respectively. For each of three image versions (original, subsampled, and small-overlapped version), a paired Student's *t*-test on ECC types reveals that the results of gradient ECC are significantly more accurate than those of intensity ECC (*P* < .01 for original version, *P* < .0005 for subsampled version and small-overlapped version) but less accurate than those of ACMI (*P* < .01 for original version, *P* < .001 for subsampled version and small-overlapped version). For each of ECC types (intensity ECC, gradient ECC, and ACMI), a one-way analysis of variance (ANOVA) on three image versions finds significant difference for intensity ECC (*P* < .001) and for gradient ECC (*P* < .005), but not for ACMI (*P* > .05). This reveals that ACMI function is much less sensitive to the reduction of resolution or overlapped area of images than intensity ECC and gradient ECC. Also, subpixel accuracy is obtained in all registrations with ACMI. As indicated by Table 2, gradient ECC performed better than intensity ECC, but it cannot achieve the optimal registration with absence of intensity information. In the fine stage of registration, the intensity information is required to further improve the registration quality. Table 2 also provides a comparison of number of iterations among intensity ECC, gradient ECC, and ACMI. As summarized by Table 2, the number of iterations was most for the intensity ECC, much more for ACMI, and least for the gradient ECC. Relative to intensity ECC and gradient ECC, ACMI provided 31.9% mean reduction but 69.8% mean increase in the number of iterations for original version, 21.4% mean reduction but 47.1% mean increase for subsampled version, and 31.9% mean reduction but 30.5% mean increase for small overlapped version, respectively. Taken together, ACMI outperforms intensity ECC in terms of accuracy and speed of registration. It can also provide a more accurate result but cost more processing time than gradient ECC. As for the fact that processing time of registration is not crucial due to highly developed computer, ACMI is preferred over gradient ECC except for a required online registration.

**Figure 7:**
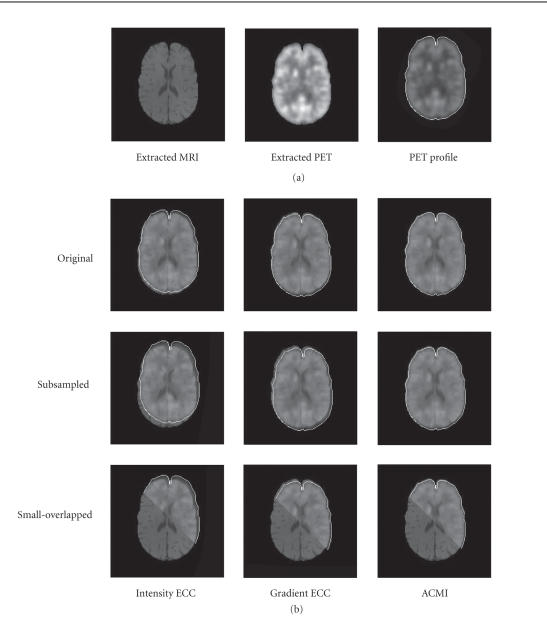
Illustration of registration results of PET/MRI T1 pair with intensity ECC, gradient ECC, and ACMI. (a) The extracted brain of MRI T1 image (left) and the 50% transparent profile of extracted brain of PET image (right) are served as the underlying and the overlying, respectively. (b) The first row: registration results of original version using intensity ECC, gradient ECC, and ACMI; the second row: the corresponding results of subsampled version; the third row: the corresponding results of small-overlapped version.

Figure 7 illustrates the registration results of three versions of MRI T1/PET image pairs with the intensity (traditional) ECC, gradient ECC, and ACMI. For the convenient display, the extracted brain of MRI T1 image (Figure 7 left of (a)) and the 50% transparent profile of extracted brain of PET image (Figure 7 right of (a)) are served as the underlying and the overlying, respectively. These examples clearly show that when the resolution or the overlapped area of images reduces, the intensity ECC and the gradient ECC can easily lead to misregistration, but the ACMI performs well (Figure 3).

## 4. DISCUSSION AND CONCLUSION

Though MI method is a well-known effective criterion for Multimodal image registration, it still has some disadvantages which often make the alignment less than optimal.

First, MI is unreliable to measure the degree of alignment between two images. MI function includes only intensity information but little spatial information of images, so it usually either produces several global maxima or presents a global maximum which does not correspond to the correct alignment. Some research introduced spatial information such as gradient-based information [6–8] or feature-based information [19–21] to improve the quality of image registration. These methods were effective but they did not took full advantage of the phase information of gradient field or the relationship between intensity images and their gradient fields.

Second, MI function is easily influenced by the intensity interpolation and presents many local maxima to trap the optimization [4, 5], leading to the failure of registration. Various high-order interpolation methods [22, 23] and global optimization algorithms [6] were introduced to reduce the influence of local maxima. But these methods are computationally expensive [24, 25]. Moreover, these methods are meaningless if the similarity measurement is unreliable [26, 27].

Third, MI is sensitive to the reduction of resolution or overlapped area of images. MI is a similarity measurement method and its reliability depends on the statistical stability of samples. The reduction of resolution or the overlapped area decreases the sample size, then deteriorates the statistical stability of samples. As a result, MI presents a poor performance for the registration of images with low resolution or small overlapped area. NMI [11] and ECC [10] were introduced to solve this problem, but no significant improvement was observed [25, 28]. They are also sensitive to the reduction of resolution or overlapped area of images.

To overcome these disadvantages of MI, we propose a technique for Multimodal image registration, namely ACMI, based on adaptive combination of intensity and gradient field mutual information. We constructed GCM from which the gradient field mutual information of original intensity images is calculated. The GCM is obtained from corresponding original images by a spherical gradient coder and includes both magnitude and phase information of gradient field of original images. The gradient field mutual information provides sufficient spatial information for the similarity measurement of images, besides it is smoother due to the relatively higher intensity uniformity of GCMs. ACMI combines the advantages of intensity ECC and gradient ECC, and adopts a coarse-to-fine and gradient-to-intensity registration strategy, so it overcomes the nonsmoothness and unreliability of traditional MI function. Results of simulated data experiments and actual registration both demonstrate that ACMI function performs better than traditional MI and it is much less sensitive to the reduction of resolution or overlapped area of two images.

According to its advantages, ACMI function is suitable for the registration of low-resolution images or impaired images. One example is the registration with multiresolution method whose object is to accelerate the registration speed without decreasing the registration accuracy and robustness. For low-resolution images, the multiresolution method with intensity ECC in fact prolongs the registration process, because the subsampling of these images deteriorates the smoothness of MI function, so that the convergence point of subsampled images is still a poor start point for the final images [29]. The ACMI can be used in multiresolution method for its insensitivity to the reduction of resolution.

In our study, the optimal value of time constant *T* was obtained using a “bootstrap” method. As shown in Figure 5, the mean registration error of each version is relatively insensitive to the changes of *T* near the optimal value, and extremely low or high *T* values can lead to relatively inaccurate registration. It is not clear whether this optimal *T* is suitable for other multimodality image pairs such as MRI/CT, MRI/SPECT. The registrations of these multimodality image pairs might present a similar pattern of “mean registration error versus time constant” to that of MRI/PET pairs (Figure 5). Extending ACMI to these multimodality image pairs will be our future work.

## Figures and Tables

**Table 1:** Mapping from gradient field of intensity image to GCM (Δ_*ρ*_ = 1/4, Δ_*θ*_ = *π*/4).

Range	[0,0.25)	[0.25,0.5)	[0.5,0.75)	[0.75,1)

[0, *π*/4)	0	8	16	24
[*π*/4, *π*/2)	1	9	17	25
[*π*/2, 3*π*/4)	2	10	18	26
[3*π*/4, *π*)	3	11	19	27
[*π*, 5*π*/4)	4	12	20	28
[5*π*/4, 3*π*/2)	5	13	21	29
[3*π*/2, 7*π*/4)	6	14	22	30
[7*π*/4, 2*π*)	7	15	23	31

**Table 2:** Accuracy and iterative number comparison among intensity ECC, gradient ECC, and ACMI.

Pair mode	Pairs	Accuracy (median/maximum mm)			Number of iterations (Mean ± SD)		
		Intensity ECC	Gradient ECC	ACMI	Intensity ECC	Gradient ECC	ACMI

*Original images*	
T1-PET	7	2.78/4.96	2.73/4.31	2.38/4.22	269.5 ± 84.8	111.3 ± 29.9	172.2 ± 44.0
T2-PET	7	1.91/6.37	1.52/5.84	1.39/5.51	288.0 ± 145.1	87.5 ± 45.8	161.7 ± 43.9
PD-PET	7	2.46/6.19	1.97/4.72	1.62/3.41	222.2 ± 108.7	93.2 ± 36.9	159.9 ± 58.6
T1 rec-PET	4	3.19/8.37	2.88/6.12	2.63/5.43	240.5 ± 92.2	102.4 ± 33.1	145.5 ± 49.7
T2 rec-PET	5	3.15/9.15	3.04/7.13	2.72/6.08	220.3 ± 97.8	93.6 ± 42.8	174.9 ± 37.3
PD rec-PET	5	3.07/8.13	2.65/7.41	2.37/6.74	224.7 ± 89.5	99.3 ± 41.9	183.1 ± 63.0
*Subsampled version*	
T1-PET	7	4.13/8.25	3.70/7.37	3.40/7.48	169.0 ± 65.0	93.7 ± 31.4	127.5 ± 36.4
T2-PET	7	4.14/9.18	3.52/6.18	3.06/5.10	189.0 ± 63.4	79.1 ± 35.9	138.8 ± 40.6
PD-PET	7	3.07/12.94	2.74/7.06	2.59/5.36	151.5 ± 63.3	82.1 ± 39.0	135.7 ± 32.8
T1 rec-PET	4	4.62/10.37	3.95/9.22	3.51/8.83	150.2 ± 59.3	88.6 ± 28.9	118.5 ± 26.3
T2 rec-PET	5	5.25/16.15	4.92/10.88	4.83/7.07	176.5 ± 59.1	89.7 ± 34.2	136.7 ± 35.6
PD rec-PET	5	4.17/11.82	3.39/8.17	3.03/6.33	144.5 ± 52.2	90.7 ± 40.6	113.7 ± 29.4
*Small-overlapped version*	
T1-PET	7	4.15/5.78	3.71/6.65	3.33/7.17	146.2 ± 93.2	94.9 ± 35.7	112.3 ± 29.9
T2-PET	7	3.34/8.37	2.44/6.07	2.20/4.94	207.5 ± 88.2	120.5 ± 61.3	153.0 ± 48.2
PD-PET	7	3.98/8.18	3.11/6.21	2.84/5.11	213.7 ± 83.1	106.8 ± 47.9	134.2 ± 51.4
T1 rec-PET	4	3.33/10.85	2.96/8.13	2.61/6.72	139.5 ± 64.1	68.6 ± 40.7	96.5 ± 43.6
T2 rec-PET	5	4.17/12.05	3.89/9.27	3.21/7.38	170.0 ± 79.3	63.9 ± 37.0	99.1 ± 46.5
PD rec-PET	5	3.39/9.10	3.30/7.58	3.16/6.91	193.0 ± 114.8	103.4 ± 58.0	133.1 ± 52.8
